# The Maintenance of Dysmyelinated Small‐Diameter Axons by 14‐3‐3s in the Central Nervous System

**DOI:** 10.1002/glia.70221

**Published:** 2026-09-09

**Authors:** Nanako Yamada, Hinako Saito, Riko Takahashi, Mio Fujisato, Yukina Hosoda, Binri Sasaki, Momona Yamada, Sakurako Abe, Chikako Hayashi, Akira Yoshimoto, Ryunosuke Ohkawa, Tomal Julian Matt, Florence M Bareyre, Stefan Berghoff, Mikael Simons, Nobuharu Suzuki

**Affiliations:** ^1^ Department of Clinical Bioanalysis and Molecular Biology, Graduate School of Medical and Dental Sciences Institute of Science Tokyo/Tokyo Medical and Dental University (TMDU) Tokyo Japan; ^2^ Department of Molecular and Cellular Biology, Graduate School of Medical and Dental Sciences TMDU Tokyo Japan; ^3^ Division of Surgical Pathology Institute of Science Tokyo Hospital Tokyo Japan; ^4^ Institute of Clinical Neuroimmunology University Hospital, LMU Munich Munich Germany; ^5^ Biomedical Center Munich, Medical Faculty, LMU Munich Munich Germany; ^6^ Munich Cluster of Systems Neurology Munich Germany; ^7^ Institute of Neuronal Cell Biology, Technical University Munich Munich Germany; ^8^ German Center for Neurodegenerative Diseases Munich Germany

**Keywords:** 14‐3‐3, axon, myelin

## Abstract

In the central nervous system, myelin formed around nerve axons by oligodendrocyte enables efficient conduction of action potentials and maintains axonal homeostasis. Therefore, in demyelinating diseases, such as multiple sclerosis (MS), axonal degeneration and loss, particularly in small‐diameter axons, are observed. Teneurin‐4 deficient (Ten‐4 ‐/‐) mice exhibit severe dysmyelination of small‐diameter axons, but survive for over a year. Here, we aimed to elucidate the mechanism of the survival of small‐diameter axons without myelin using Ten‐4 ‐/‐ mice. An immunohistochemical analysis showed abnormally diffused and intense staining of neurofilament. Further, the immunohistochemical signal of nonphosphorylated neurofilament and amyloid precursor protein was higher in Ten‐4 ‐/‐ mice. An electron microscopy analysis unexpectedly revealed that small‐diameter axons in Ten‐4 ‐/‐ mice survived at the age of 1 year, despite the absence of compact myelin. To elucidate the molecular mechanism, we performed a proteomics analysis in the Ten‐4 ‐/‐ tissue by mass spectrometry. The result indicated that four of the seven 14‐3‐3 isoforms were highly expressed in Ten‐4 ‐/‐ small‐diameter axons. Finally, when the function of 14‐3‐3s in Ten‐4 ‐/‐ mice was inhibited by 14‐3‐3 inhibitors, BV02 and difopein, the progression of small‐diameter axon damage was observed. Altogether, 14‐3‐3s are among the pro‐survival factors in dysmyelinated small‐diameter axons. Our findings may be useful for better understanding the pathogenesis of demyelinating diseases, such as MS, as well as for the development of new treatments.

## Graphical abstract

- Dysmyelinated small‐diameter axons are maintained in teneurin‐4 deficient mice at the age of 1 year, while axonal damage is observed.
- 14‐3‐3s are highly expressed and suppress the progression of the damage in these axons.

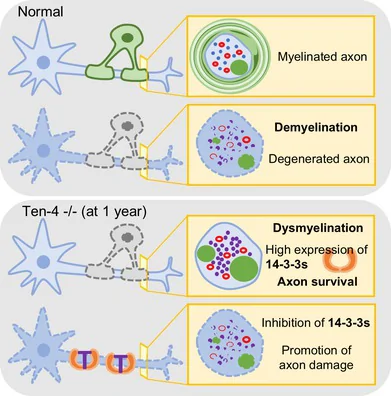

## 1 Introduction

In the central nervous system (CNS), myelin is formed by oligodendrocytes (OLs) and ensheathes nerve axons with the structure of multiple layers of sheet‐like OL plasma membrane. Myelin enables the saltatory conduction of nerve action potentials and plays a critical role in the maintenance of axonal homeostasis through supplying monocarboxylates, such as lactate and pyruvate (Lee et al. 2012). In multiple sclerosis (MS), the most typical demyelinating disease, many patients exhibit the relapsing–remitting form with repeated demyelination and remyelination in the CNS, followed by the secondary progressive form with axon degeneration due to the chronic demyelination. Severe symptoms such as visual impairment, autonomic neuropathy, and muscle weakness are caused, but there are few effective treatments for the secondary progressive form (Kuhlmann et al. 2023).

The axonal cytoskeletons are mainly composed of microtubules (MTs), neurofilaments (NFs), and actin filaments, which are aligned along the axon's longitudinal axis (Kevenaar and Hoogenraad 2015). These cytoskeletons are well organized in myelinated axons. However, demyelinated axons represent the decrease in NF spacing due to their dephosphorylation and the fragmentation of NFs (Dutta and Trapp 2011). Also, amyloid precursor protein (APP) is widely used as an axonal injury/damage marker in many studies with humans and rodents (Gentleman et al. 1995; Kuhlmann et al. 2002; Höflich et al. 2016). APP is rapidly transported from the neuronal cell body to the axonal terminal and is hardly detected by immunohistochemical staining in healthy axons (Höflich et al. 2016). APP forms a complex with kinesin‐I, a MT motor protein, and the deletion of the kinesin light chain, which possesses the binding domain to APP, decreased the axonal transport of APP (Kamal et al. 2000). When axons are injured, disintegration and reduction of the cytoskeletal elements including MTs are observed (Dutta and Trapp 2011). This likely induces an impairment of axonal transport of APP; thereby, disruption of the axonal transport leads to the accumulation of APP, which is detectable by immunohistochemistry, in the demyelination model with cuprizone (Rühling et al. 2019). During demyelination, the other axoplasm components are also changed. The number and size of mitochondria are increased at the demyelination (Mahad et al. 2009). At the early stage of demyelination, ATP production from mitochondria is highly required for the excess activation of Na^+^/K^+^ ATPases for the efflux of Na^+^ from axoplasm. However, the ATP production gradually becomes insufficient for the maintenance of Na^+^/K^+^ ATPase's function, and Na^+^/Ca^2+^ exchangers are activated on behalf of Na^+^/K^+^ ATPases at the late stage. The influx and accumulation of Ca^2+^ induce activation of proteolytic enzymes, such as calpain, which degrade axonal components including cytoskeletal and mitochondrial proteins. Eventually, axons die and disappear (Trapp and Stys 2009). In addition, it is interesting that the degeneration and loss of axons in MS are predominantly observed in small‐diameter axons, which are typically located in the corticospinal tract (CST) and fasciculus gracilis (FG) of the spinal cord (DeLuca et al. 2004). However, the detailed mechanism has not been clarified.

Although various animal models have been used to elucidate the mechanism of axon maintenance by myelin, they are not perfectly suitable for studying it. For example, *shiverer* mice that lack the expression of myelin basic protein (MBP), an essential component of myelin, fail normal myelination and develop severe tremors (Chernoff 1981). However, the change of the axons after the defect of myelination cannot be observed in the long term because of the short life span (~100 days). In contrast to *shiverer*, mice deficient in proteolipid protein (PLP), which is a major myelin protein as well as MBP, display axonal degeneration, whereas these axons are fully myelinated (Griffiths et al. 1998). Similarly, in the knockout mice of 2′, 3′‐cyclic nucleotide 3′‐phosphodiesterase (CNP), an OL‐specific membrane‐associated protein, axonal degeneration emerges despite forming myelin normally (Lappe‐Siefke et al. 2003). It is beneficial to study the mechanism of axon maintenance, especially with its small‐diameter, without myelin in the long term, if a mutant mouse line that shows dysmyelination and survives at least a year is available.

Teneurin‐4 (Ten‐4), a Type II transmembrane protein, is expressed in both OLs and neurons and promotes cell adhesion between them (Hayashi, Suzuki, Mabuchi, et al. 2020). In our previous study, we reported the prominent dysmyelination in the small‐diameter axon regions, the CST and FG, of Ten‐4 deficient (−/−) mice (Suzuki et al. 2012). Ten‐4 ‐/‐ mice further develop severe phenotypes such as tremors in their hindlimbs; however, they survive longer than a year: some of them survive for more than 2 years. This mutant mouse line must be useful for analyzing small‐diameter axons with dysmyelination in the long term.

In this study, we therefore aimed to elucidate the molecular mechanism in the maintenance and/or degeneration of small‐diameter axons without myelin in the CNS by using Ten‐4 ‐/‐ mice. Our results indicated that the small‐diameter axons showed an increase in immunopositivity of axonal damage markers. However, these axons did not disappear and were maintained for more than a year. In a proteomics analysis, we found that four of the seven isoforms of 14‐3‐3, which are multifunctional proteins that regulate various cellular signaling including the suppression of apoptosis via the mitochondrial permeability (Stevers et al. 2018). Finally, inhibition experiments of 14‐3‐3s revealed the pro‐survival function of 14‐3‐3s in the small‐diameter axons. Our findings should be useful for better understanding of the mechanism in the maintenance of dysmyelinated small‐diameter axons and for the development of possible therapeutic reagents for MS and other myelin‐related diseases.

## 2 Materials and Methods

### 2.1 Animals

The Ten‐4 ‐/‐ mouse line (*furue*) was kindly provided by Dr. Yoshihiko Yamada from NIDCR, NIH. Wild‐type (WT) and Ten‐4 ‐/‐ littermates at postnatal day (P) 7, 7 weeks, 1 year, and 1 year and 7 months were used for experiments. All procedures for genetic recombination experiments and animal experiments were approved by the internal committees of the Institute of Science Tokyo/Tokyo Medical and Dental University (TMDU) (Approval No: G2024‐004C3 and A2025‐042C), followed the Animal Care Standards of the Institute of Science Tokyo/TMDU in the management and handling of experimental animals, and complied with the ARRIVE guidelines. An equal number of male and female animals was used for the experiments.

### 2.2 Tissue Sections

For the preparation of frozen tissue sections, WT and Ten‐4 ‐/‐ mice were anesthetized with an injection of the mixed agent of medetomidine hydrochloride (ZENOAQ) (0.75 mg/kg), midazolam (NIG) (4 mg/kg), and butorphanol tartrate (Meiji Seika Pharma) (5 mg/kg). The anesthetized mice were perfused with phosphate‐buffered saline (PBS) and then fixed with 4% paraformaldehyde (PFA) (FUJIFILM Wako Pure Chemical) in PBS. Spinal cords were dissected out and postfixed in 4% PFA in PBS overnight at 4°C. Fixed tissues were placed in 15% sucrose in PBS overnight at 4°C, and then 30% sucrose in PBS for 2 days at 4°C. The tissues were embedded in O.C.T. compound (Sakura Finetek) on dry ice. After that, the tissues were sliced at 8‐μm thickness with cryostat (Leica LCM).

### 2.3 Immunohistochemistry

Tissue sections were fixed with 4% PFA in PBS for 10 min at room temperature (RT) and were washed with PBS. Then, sections were permeabilized with 0.1% Triton X‐100 (Merck) diluted in PBS or antigen retrieval was performed with Target Retrieval Solution (Agilent Technologies) using a microwave. The tissue sections were then blocked with Power Block Universal Blocking Reagent (BioGenex Laboratories) for 1 h at RT. After blocking, primary antibodies, mouse anti‐NF (1:200; Merck), rabbit anti‐NF (1:500; Merck), mouse anti‐NF‐H, Nonphosphorylated (SMI‐32; 1:2500; BioLegend), rabbit anti‐APP (1:1000; Merck), rabbit anti‐Iba1 (1:250; FUJIFILM Wako Pure Chemical), rabbit anti‐glial fibrillary acidic protein (GFAP) (1:1000; Abcam), mouse anti‐pan 14‐3‐3 (1:50; Santa Cruz Biotechnology), and rabbit anti‐green fluorescent protein (GFP) (1:200; MBL) in 1% bovine serum albumin (BSA) in PBS were incubated overnight at 4°C. Next, tissue sections were washed with PBS and incubated with secondary antibodies, goat anti‐mouse IgG‐Alexa 488 (1:250; ThermoFisher Scientific), goat anti‐mouse IgG‐Alexa 594 (1:250; ThermoFisher Scientific), goat anti‐rabbit IgG‐Alexa 488 (1:250; ThermoFisher Scientific), and goat anti‐rabbit IgG‐Alexa 594 (1:250; ThermoFisher Scientific) for 50 min at RT. After washing with PBS, the immunostained samples were mounted with Mounting Medium (Ibidi) containing DAPI solution (1:5000; Dojindo Laboratories) diluted in PBS.

For the quantification of immunohistochemically positive area, we set a threshold of the immunofluorescence intensity in a pair of a sample group and a control group in each measurement using the Image J software. The threshold was determined as an intensity just below a saturation level of its fluorescent signal in each measurement analysis. And then, the positive area whose fluorescent signal was higher than the threshold was divided by the total area of the region (see Figure S1 for the determination of the regions). This ratio is the percentage of the positive area in each graph data.

### 2.4 Electron Microscopy (EM) Analysis

For the representative images in Figure 5, WT and Ten‐4 ‐/‐ mice were anesthetized as described in the paragraph of Tissue sections. And then, they were perfused with PBS and fixed with 2.5% glutaraldehyde in 0.1 M phosphate buffer (PB). Spinal cords were dissected out and fixed in 2.5% glutaraldehyde in 0.1 M PB for 2 h at 4°C. After washing with 0.1 M PB, tissues were postfixed in 1% osmium tetroxide buffered with 0.1 M PB for 2 h at 2°C. Transmission EM (TEM) analysis was performed by Tokai Electron Microscopy Inc. For the quantification analyses in Figure 5, we prepared frozen sections of spinal cords as described in the paragraph of Tissue sections. Scanning EM (SEM) analyses were performed by Ochanomizu research facility in Institute of Science Tokyo. Representative SEM images for the quantification analyses are shown in Figure S2. Image J was used for the analyses. The criterion for identifying axons was a cytoplasm with a circular or oval shape, whose longest diameter was more than 1 μm, and containing NFs, MTs, and mitochondria. A cytoplasm like glial process with microvilli that were typically 50–60 nm thickness and contained a moderately dense cytoplasm, in which a few fine filaments, and a larger foot‐like shaped glial process were excluded (Hildebrand et al. 1993; Mey et al. 2025). Six to eight hundred axons from three individual mice were analyzed for the quantifications.

For the EM analyses in Figures 7 and 8, we prepared frozen sections of spinal cords as described in the paragraph of Tissue sections. TEM analysis was performed by Ochanomizu research facility in Institute of Science Tokyo for the representative images. For the quantification analyses, SEM analyses were performed by Ochanomizu research facility in Institute of Science Tokyo. Representative SEM images for the quantification analyses are shown in Figure S2. Image J was used for the analyses. The criterion for identifying axons was a cytoplasm with either an existence of mitochondria or more than 1 μm longest diameter. One hundred axons from three individual mouse CST areas were randomly selected and processed using a threshold that allowed distinction of mitochondria and axonal plasma, followed by binarization. The axonal borders were selected and the areas of axons were measured, and then, the areas of mitochondria and MTs within the axons were measured using the function of particle analysis. The areas of total mitochondria and total MTs were respectively divided by the axon areas to calculate the percentage of their occupying areas in axons.

### 2.5 Shotgun Mass Spectrometry Analysis

To euthanize 1‐year‐old mice, cervical dislocation was performed. The spinal cords of mice were dissected out. Then white matter dorsal columns (DCs) were isolated from the spinal cords and lysed using MPEX PTS Reagents for MS (GL Sciences). Shotgun mass spectrometry and label‐free quantification using the search engine SequestHT were performed by the Ochanomizu research facility in the Institute of Science Tokyo.

### 2.6 Western Blotting

To euthanize 1‐year‐old mice, cervical dislocation was performed. The DC tissues from WT and Ten‐4 ‐/‐ mice were used as samples. Procedures were followed according to the condition of the previous study (Hayashi, Suzuki, Mabuchi, et al. 2020). Primary antibodies were mouse anti‐pan 14‐3‐3 and mouse anti‐β‐actin (Fujifilm Wako Pure Chemicals Corporation). Secondary antibodies were anti‐mouse IgG antibody‐HRP (Cell Signaling Technology). As detection reagents, ImmunoStar LD (Fujifilm Wako Pure Chemicals Corporation) and ECL Prime Western Blotting Detection Reagent (cytiva) were used for pan 14‐3‐3 and β‐actin, respectively.

### 2.7 Plasmid Construction and Preparation of Adeno‐Associated Virus (AAV)

The plasmid (pSCM‐138) containing the cDNA sequence of difopein, which is the artificial peptide (Masters and Fu 2001), was kindly provided by Dr. Haian Fu from Emory university. For the vector, the AAV expression plasmid containing the synapsin‐1 promoter, which is specifically driven in neurons (pAAV‐hSyn‐EGFP) (Addgene), was used. We performed PCR to amplify the difopein fragment as an insert with the primers (forward: 5′‐GCATGGACGAGCTGTACAAGTCCGGACTCAGATCCGAA‐3′; reverse: 5′‐TAAGCTTGATATCGAATTCGCAGAATTCGGCTTATCTAGA‐3′). For vector amplification, we used the following primers (forward: 5′‐GAATTCGATATCAAGCTTATCG‐3′; reverse: 5′‐CTTGTACAGCTCGTCCATGC‐3′). The insert and the vector obtained by the PCRs were assembled using the NEBuilder HiFi DNA Assembly Cloning Kit (New England Biolabs). These plasmids were amplified in the culture of *Escherichia* *coli* competent cell line DH5α (TOYOBO) and purified using the EndoFree Plasmid Maxi Kit (QIAGEN).

For preparation of AAV‐PHP.S containing pAAV‐hSyn‐EGFP or pAAV‐hSyn‐EGFP‐Difopein, the plasmids were transfected into HEK293 cells using polyethylenimine (Polysciences Inc.). After 5 days, cells were collected and centrifuged at 1000*g* for 10 min at 4°C. Supernatant was filtered through a 0.45 μm polyethersulfone membrane and was added 40% polyethylene glycol (Merck) solution to the supernatant. The mixture was stirred slowly for 1 h at 4°C, then left overnight at 4°C. On the next day, the mixture was resuspended by adding 0.001% pluronic F68 (ThermoFisher Scientific) and 200 mM NaCl in PBS and sonicated for 1 s (50% amplitude) four times with 15‐min interval on ice between each pulse. Then, we pelleted cell debris by centrifugation at 3220 *g* for 15 min at 4°C and transferred the cleared lysate to a new tube. It was left overnight at 4°C. We combined the virus and cell pellet into one tube and centrifuged at 2820*g* for 15 min at 4°C. Then, we added PBS containing 200 mM NaCl and 0.001% F68 to the tube. After that, 50 units of Benzonase Nuclease (Merck) per milliliter of viral suspension in PBS were added and incubated for 45 min at 37°C. Next, we prepared a barrel with a density gradient for ultracentrifugation and pipetted the lightest gradient first, and then, pipetted the heavier gradient at the bottom with a 10 mL syringe connected to a long needle, to build up the density gradient. We then topped up the gradient with the virus and centrifuged them at 58,400*g* for 2 h and 25 min at 18°C under vacuum conditions. Using a 16G 1½ needle and 5 mL syringe, we poked into the barrel and slowly extracted the virus from the transparent layer. Then, we screwed a 10 mL LL syringe on a 0.22 μm filter, placed it on the equilibrated Amicon filter (Merck), and removed the plunger. The syringe filled with little over 10 mL of PBS was pushed out until 10 mL to fill the filter. The virus was placed at the bottom of the 10 mL syringe using the needle. The syringe was emptied through the filter by pushing the plunger all the way down. After centrifugation at 3000*g* for 10 min at 4°C, we discarded the flow‐through, filled the filter with PBS, and mixed the solution in the Amicon filter with a 200 μL pipette without touching the filter surface. It was repeated without adding PBS to concentrate the viral suspension to ~250 μL and then all viruses were pipetted into a 0.5 mL protein LoBind Tube (Eppendorf). For titer measurement, the quantitative PCR method was performed.

### 2.8 14‐3‐3 Inhibition Experiments

For inhibition experiments with BV02 (Merck), BV02 was dissolved in dimethylsulfoxide (DMSO) (FUJIFILM Wako Pure Chemical) at 50 mg/mL. It was diluted 12.5 times with PBS and 100 μL of the diluted BV02 was administered intraperitoneally to Ten‐4 ‐/‐ mice at a final concentration of 20 mg/kg. The same injection was performed every day for 7 days. As a control, the same amount of diluted DMSO with PBS was administered intraperitoneally to Ten‐4 ‐/‐ mice. For inhibition experiments with difopein, the AAV‐PHP.S containing difopein or the control, which is described in the paragraphs of Plasmid construction and preparation of AAV, was intravenously injected into the retro‐orbital plexus of Ten‐4 ‐/‐ mice at 1 × 10^12^ vg, after they were anesthetized as described in the paragraph of Tissue sections. After the AAV injections, atipamezole (ZENOAQ) (0.75 mg/kg), an antagonist for the mixed anesthesia, was intraperitoneally injected. The mice were housed for 3 weeks to let the injected AAVs infect as reported (Chan et al. 2017).

### 2.9 Statistical Analyses

All the experiments were independently performed at least three times (three individuals). The statistics of the experimental results were analyzed relative to the controls. Student's *t*‐test was used for the analyses in the experiments with two groups. Microsoft Excel was used for the statistical analyses. The statistical significance was defined as *p < 0.05, **p < 0.01, ***p < 0.001.

## 3 Results

### 3.1 Axonal Damage or Degeneration in the Small‐Diameter Axon Regions in Ten‐4 ‐/‐ Mice

**FIGURE 1:**
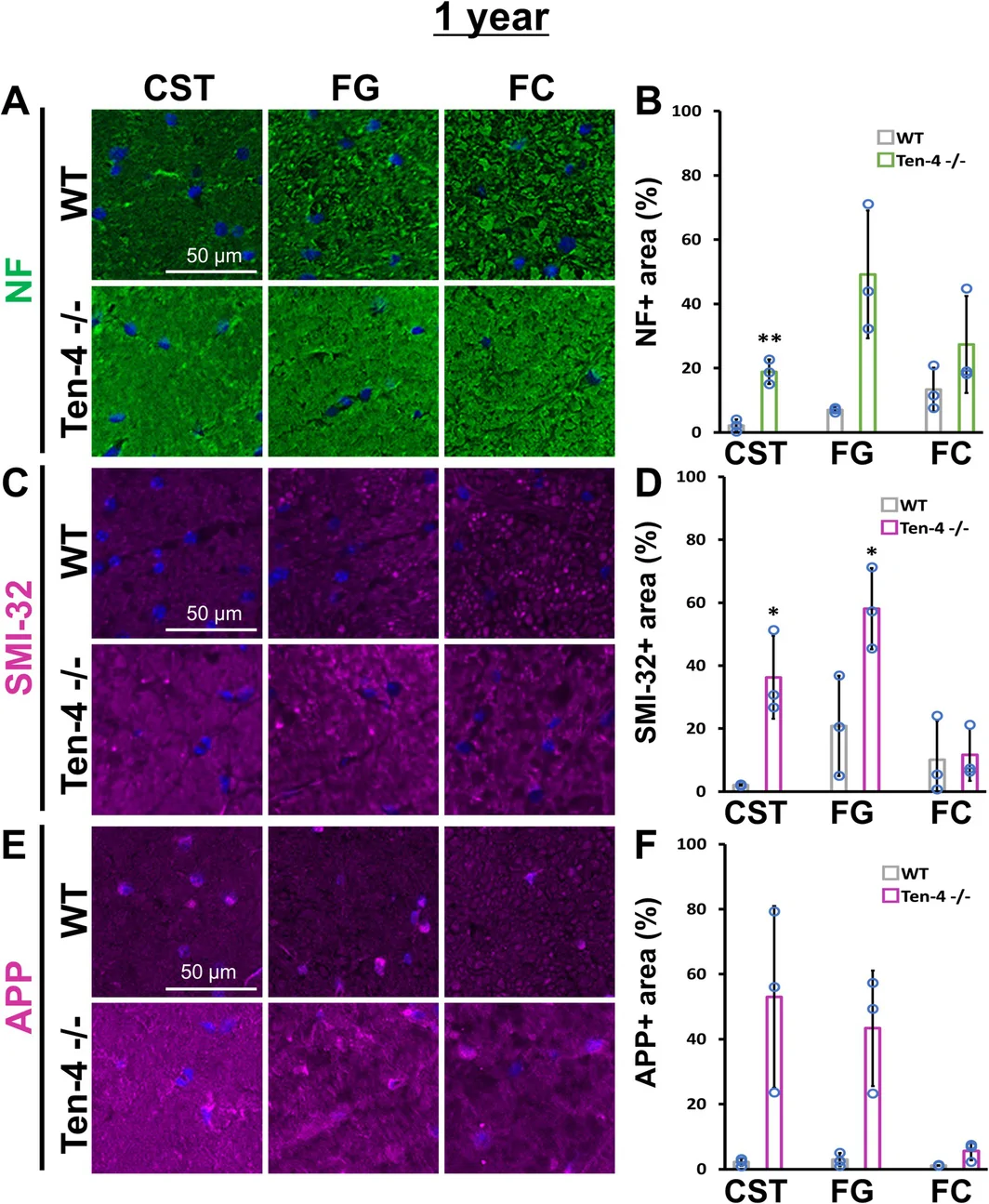
Axonal damage in the small‐diameter axon regions in 1‐year‐old Ten‐4 ‐/‐ mice. Immunohistochemical images of NF (A), nonphosphorylated NF (SMI‐32) (C), and APP (E) in DCs of WT and Ten‐4 ‐/‐ spinal cords. DAPI was used to stain nuclei. NF was diffusely stained in the CST and FG of Ten‐4 ‐/‐ mice. In addition, most of these axons in the Ten‐4 ‐/‐ CST and FG were positive for SMI‐32 and APP. Scale bar: 50 μm. Quantifications of NF‐positive (B), SMI‐32‐positive (D), and APP‐positive (F) areas in each region. Three individual mouse tissues were analyzed (*p < 0.05, **p < 0.01, Student's *t*‐test, error bars represent mean ± SD).

To characterize dysmyelinated small‐diameter axons, we performed an immunohistochemical analysis using 1‐year‐old mouse spinal cords. The DC of the spinal cord consists of the CST, FG, and fasciculus cuneatus (FC) in mice (Figure S1). In morphological characteristics, small‐diameter axons are dominantly located in the CST and FG, and partially in the FC, while larger‐diameter axons are abundant in the FC. As mentioned above, APP proteins are accumulated and become immunopositive at the axonal injury after demyelination. Also, in many studies, the antibody SMI‐32 against non‐phosphorylated NF is used as a marker for axonal damage and/or degeneration in demyelinated axons (Rosenfeld et al. 1987; Mancardi et al. 2001; Dziedzic et al. 2010). Therefore, we performed immunohistochemistry of NF, SMI‐32, and APP. As a result, it showed intense and diffused staining patterns of NF in the small‐diameter axon regions of the CST and FG, and partially in the FC, in Ten‐4 ‐/‐ mice, compared with WT littermates (Figure 1A). There was a statistical difference between WT and Ten‐4 ‐/‐ mice in the intensity of NF staining in the CST (Figure 1B). In addition, most of the NFs, which were diffusely stained, in small‐diameter axons of Ten‐4 ‐/‐ tissues were positive for SMI‐32 and APP (Figure 1C,E). The SMI‐32 intensity gave statistical differences in the CST and FG, whereas that of APP did not reach statistical significance due to the dispersion between the individuals (Figure 1D,F). These results suggested that axonal damage or degeneration occurred preferentially in small‐diameter axons in the Ten‐4 ‐/‐ tissues.

**FIGURE 2:**
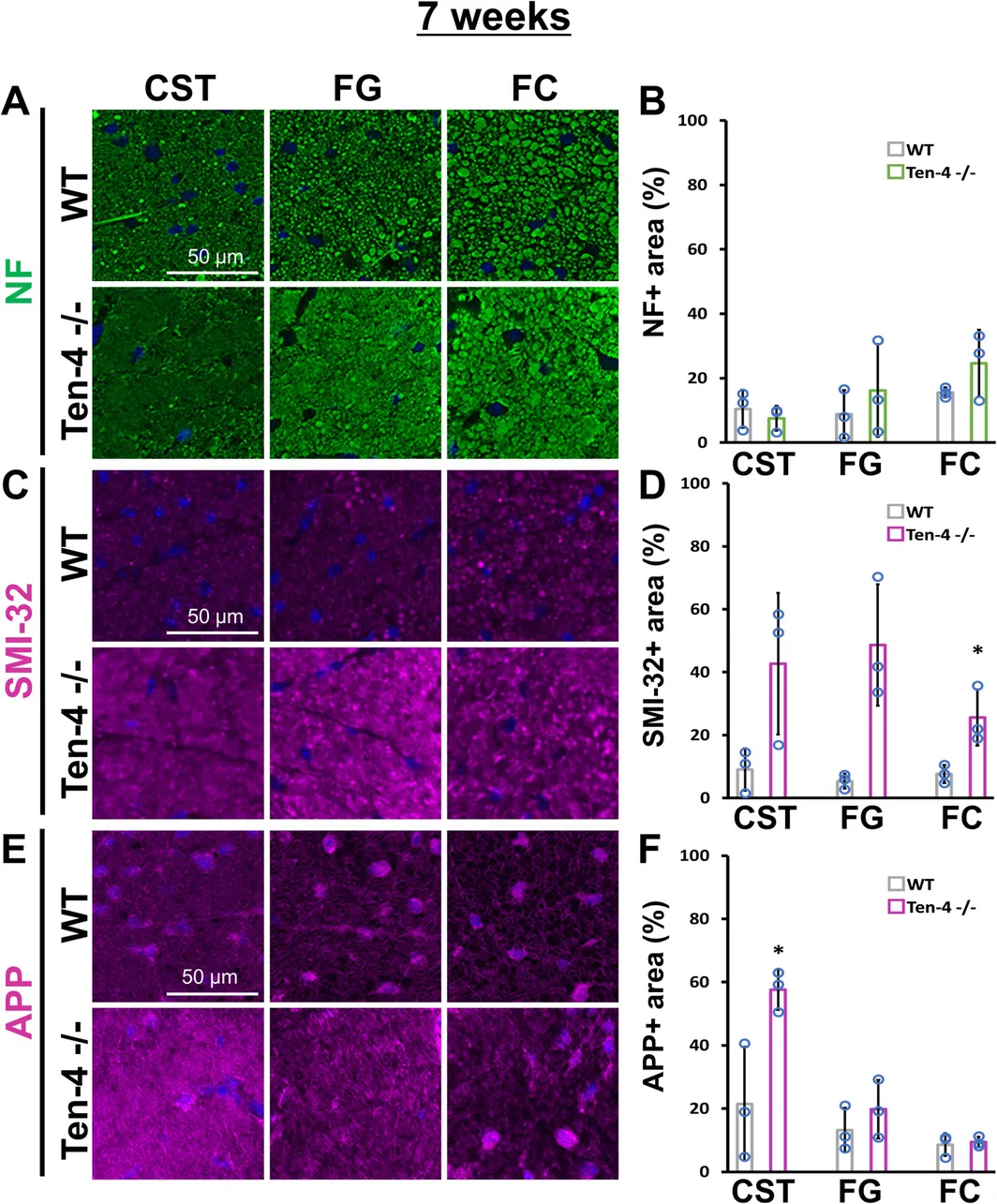
Axonal damage in the small‐diameter axon regions in 7‐week‐old Ten‐4 ‐/‐ mice. Immunohistochemical images of NF (A), non‐phosphorylated NF (SMI‐32) (C), and APP (E) in DCs of WT and Ten‐4 ‐/‐ spinal cords. DAPI was used to stain nuclei. NF was diffusely stained in the CST and FG of Ten‐4 ‐/‐ mice. Most of NFs in these axons were non‐phosphorylated. APP immunoreactivity was increased in the Ten‐4 ‐/‐ CST. Scale bar: 50 μm. Quantifications of NF‐positive (B), SMI‐32‐positive (D), and APP‐positive (F) areas in each region. Three individual mouse tissues were analyzed (*p < 0.05, Student's *t*‐test, error bars represent mean ± SD).

**FIGURE 3:**
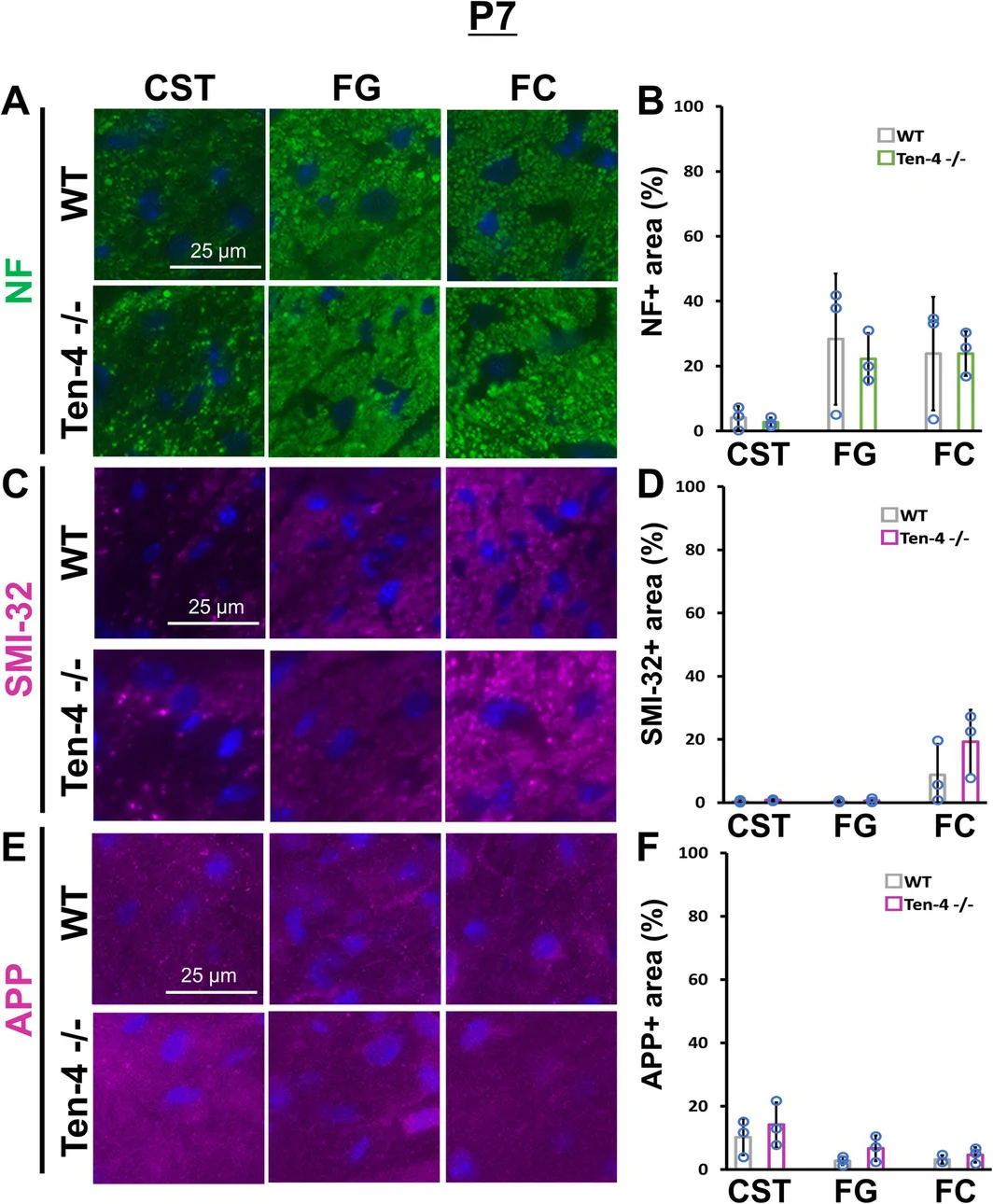
Normal axon formation in the dorsal spinal cord in P7 Ten‐4 ‐/‐ mice. Immunohistochemical images of NF (A), nonphosphorylated NF (SMI‐32) (C), and APP (E) in DCs of WT and Ten‐4 ‐/‐ spinal cords. DAPI was used to stain nuclei. No significant differences between WT and Ten‐4 ‐/‐ mice were observed. Scale bar: 25 μm. Quantifications of NF‐positive (B), SMI‐32‐positive (D), and APP‐positive (F) in each region. Three individual mouse tissues were analyzed (Student's *t*‐test, error bars represent mean ± SD).

To examine when the damage/degeneration began, we then performed the immunohistochemistry of NF, SMI‐32, and APP at the age of 7 weeks, around the time when CNS myelination is completed in WT mice. In the Ten‐4 ‐/‐ CST and FG, NF was diffusely stained (Figure 2A), but the diffused staining was less than that in the 1‐year‐old tissues. The intensity of NF staining was normal in Ten‐4 ‐/‐ mice (Figure 2B). The signal of SMI‐32 was higher in Ten‐4 ‐/‐ tissues, while a statistical significance was obtained in the FC, but not in the CST and FG due to the dispersion between the individuals (Figure 2C,D). The high immunopositive signal of APP was observed only in the Ten‐4 ‐/‐ CST (Figure 2E,F). APP‐positive cells, which were presumably OLs, were often observed in WT tissues as previously reported, though they were rarely found in Ten‐4 ‐/‐ tissues as OLs are significantly reduced in Ten‐4 ‐/‐ spinal cord at the age of 7 weeks (Hayashi, Suzuki, Takahashi, et al. 2020; Sasmita et al. 2024). We next tested the same combination of immunohistochemistry at P7, when OL myelination is initiated in WT. We observed that there were no differences between WT and Ten‐4 ‐/‐ mice in intensity and staining patterns of NF, SMI‐32, and APP at P7 (Figure 3A–F), albeit SMI‐32 staining does not function as a marker for the degeneration at this age since the phosphorylation of NF occurs dependent on myelination of axons. From these results, axons were normally formed before the onset of myelination; however, degenerated small‐diameter axons were increased, plausibly because of the impairment of OL myelination in Ten‐4 ‐/‐ mice.

**FIGURE 4:**
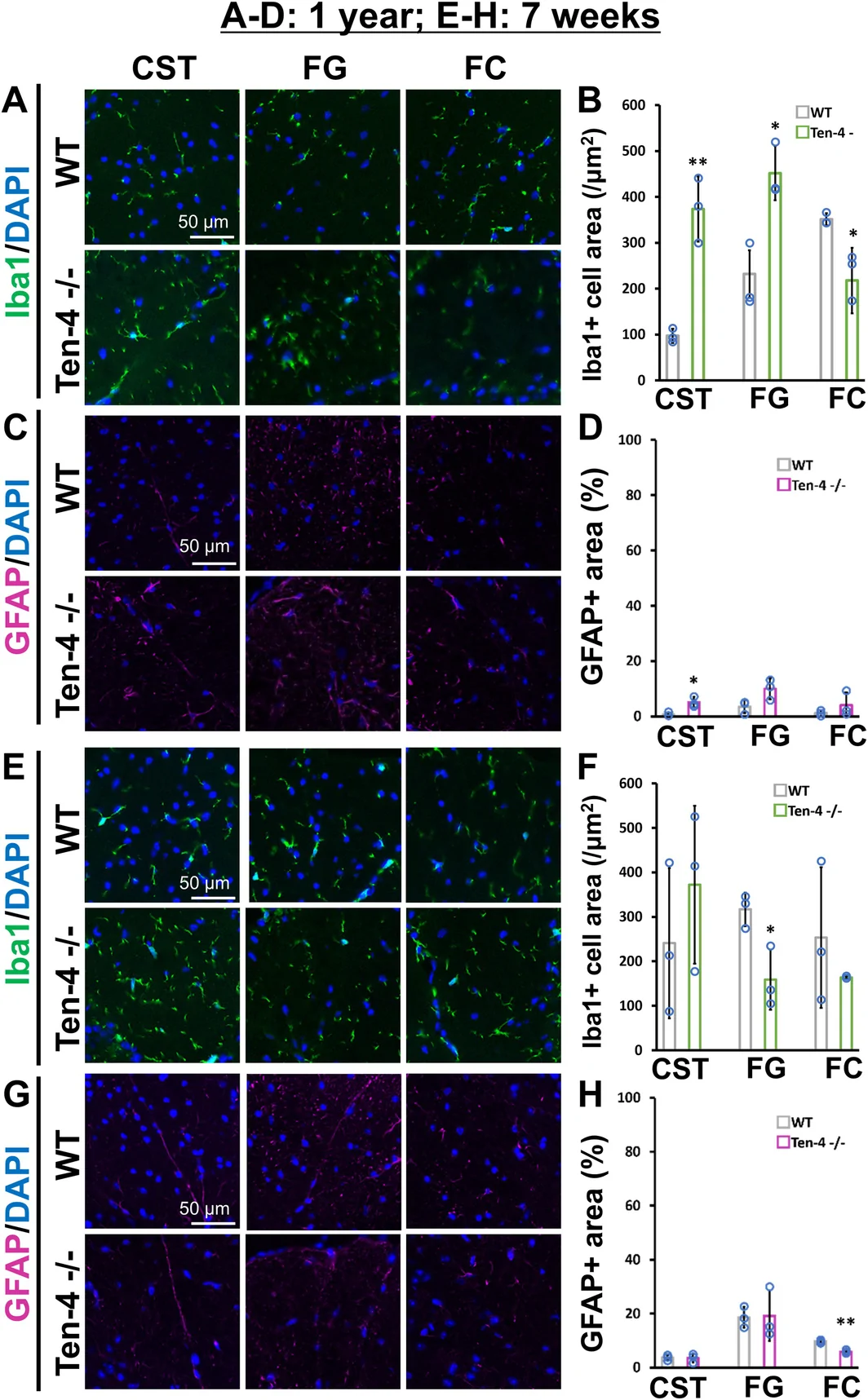
Glial reaction in the small‐diameter axon regions in 1‐year‐old Ten‐4 ‐/‐ mice. Immunohistochemical images of activated microglia (Iba1) (A): 1‐year; (E): 7‐weeks; and astrocyte (GFAP) (C): 1‐year; (G): 7‐weeks; in DCs of WT and Ten‐4 ‐/‐ spinal cords. DAPI was used to stain nuclei. Activation of microglia and increase in astrocyte processes were observed in the CST and FG of 1‐year‐old Ten‐4 ‐/‐ mice. Scale bar: 50 μm. Quantifications of the size of Iba1‐positive cell (B): 1‐year; (F): 7‐weeks; and GFAP‐positive area (D): 1 year; (H): 7 weeks; in each region. Three individual mouse tissues were analyzed (*p < 0.05, **p < 0.01, Student's *t*‐test, error bars represent mean ± SD).

In addition, to determine whether Ten‐4 ‐/‐ tissues exhibit activation of microglia and astrocytic gliosis, which often occur with axonal damage/degeneration, we performed immunohistochemistry of Iba1 and GFAP, markers for microglia and astrocytes, respectively. As a result, the signal of Iba1 and GFAP expression was elevated by the increase of cell number, the enlargement of cell bodies, and/or the extension of cellular processes in the CST and FG in Ten‐4 ‐/‐ mice at the age of 1 year (Figure 4A–D). The increases were observed in neither Iba1 nor GFAP staining at 7 weeks (Figure 4E–H). These glial reactions in the aged Ten‐4 ‐/‐ tissues were consistent with the results of immunohistochemical analyses of NF, SMI‐32, and APP.

### 3.2 Maintaining of Dysmyelinated Small‐Diameter Axons in Ten‐4 ‐/‐ Mice

**FIGURE 5:**
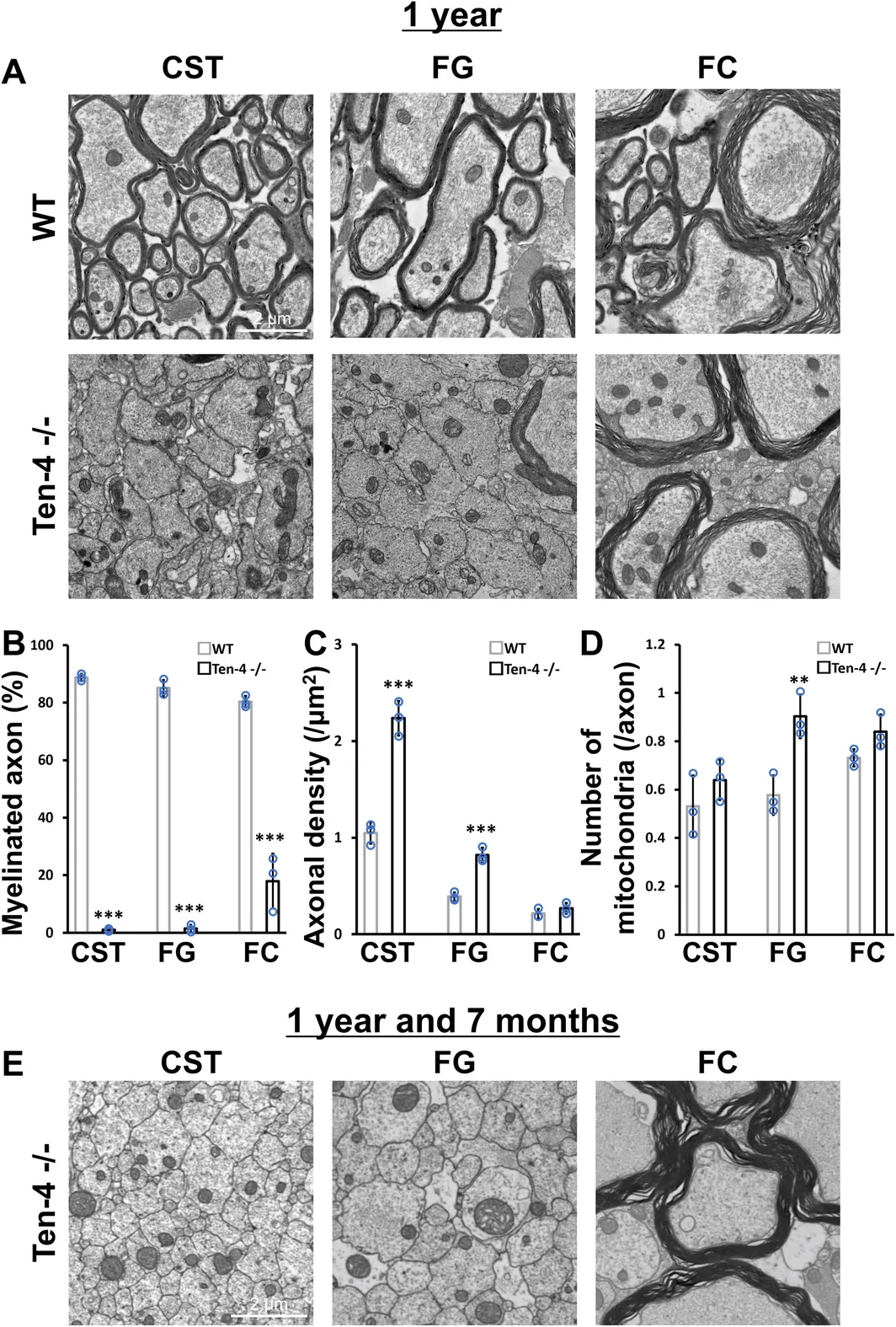
Maintaining of dysmyelinated axons with small‐diameters. (A) TEM images of myelin and axon structures at the age of 1 year. The small‐diameter axons did not disappear but were maintained in Ten‐4 ‐/‐ mice. Scale bar: 2 μm. Quantifications of myelinated axon (B), axonal density (C), and mitochondria number per axon (D) in each region of 1‐year‐old mice. Three individual mouse tissues were analyzed (**p < 0.01, ***p < 0.001, Student's *t*‐test, error bars represent mean ± SD). (E) TEM images of myelin and axon structures at the age of 1 year and 7 months. Dysmyelinated small‐diameter axons were still maintained in Ten‐4 ‐/‐ mice. Scale bar: 2 μm.

To observe the ultrastructure of small‐diameter axons associated with damage or degeneration, we carried out an EM analysis using 1‐year‐old mouse spinal cords. As in our previous study (Suzuki et al. 2012), we observed dysmyelination of small‐diameter axons in Ten‐4 ‐/‐ tissues (Figure 5A,B). Due to the dysmyelination, axonal densities were higher in the CST and FG (Figure 5C). However, surprisingly, these dysmyelinated small‐diameter axons did not disappear but were maintained in Ten‐4 ‐/‐ tissues, while the dots signal of NFs increased compared with WT (Figure 5A). The increase of NF dots was presumably due to fragmentation of NFs, which was more detectable with the immunohistochemical analysis (Figure 1A,B). We were rarely able to find typical degenerated morphologies, such as axonal swelling and organelle accumulation. In addition, we counted the number of mitochondria per axon, and the result showed that it was significantly elevated in the FG region of Ten‐4 ‐/‐ mice (Figure 5D). Moreover, we found that the dysmyelinated small‐diameter axons were still maintained at the more aged stage, 1 year and 7 months, in Ten‐4 ‐/‐ mice (Figure 5E). Similar trends were already observed at the age of 7 weeks, a stage of the completion of WT myelination (Figure S3). These data indicated that Ten‐4 ‐/‐ small‐diameter axons were damaged with the increase of the markers' immunoreactivity but not degenerated with the typical morphological abnormalities. As a result, these axons survived without myelin in the long term and there must be a molecular mechanism of the positive regulation in the maintenance of dysmyelinated small‐diameter axons in Ten‐4 ‐/‐ mice.

### 3.3 Exploring Key Molecules for Maintaining Small‐Diameter Axons

To explore the proteins that are highly expressed and regulate the maintenance of dysmyelinated small‐diameter axons, we performed a comprehensive proteomics analysis using 1‐year‐old mouse tissues. We dissected out the DCs and carried out a shotgun mass spectrometry. We then performed a label‐free quantification of the protein expression between WT and Ten‐4 ‐/‐ tissues using the mass spectrometry result. As a result, there were 37 proteins whose expression levels were significantly different between WT and Ten‐4 ‐/‐ tissues. Six and thirty‐one proteins were obtained as downregulated and upregulated proteins in Ten‐4 ‐/‐ DC, respectively. The six proteins reduced in Ten‐4 ‐/‐ mice were OL specific proteins (Table 1). This result made sense since OLs were significantly decreased in the Ten‐4 ‐/‐ DC tissue (Suzuki et al. 2012; Hayashi, Suzuki, Takahashi, et al. 2020). On the other hand, the proteins listed in Table 2 were upregulated in the Ten‐4 ‐/‐ DC. NFs and the other axonal cytoskeletal proteins were detected in large amounts (Table 2). Probably, fragmentations of these proteins due to the degradation enhanced their detectability in this experimental condition. Thus, the increase of these cytoskeletal proteins presumably occurred as a secondary effect of the axonal damage. Also, several proteins in the pathways for ATP synthesis, such as ATP synthase subunits and phosphopyruvate hydratase, were listed (Table 2). This result suggested that mitochondrial metabolism and glycolysis were more activated in the maintenance of dysmyelinated axons in Ten‐4 ‐/‐ mice. This correlated with the increase of mitochondria in the Ten‐4 ‐/‐ small‐diameter axons. Apart from these proteins, we searched for more upstream regulatory proteins for the energy metabolism and/or pro‐survival pathways and found that 14‐3‐3 protein isoforms (*β*, *γ*, *ε*, and *ζ*) were listed as upregulated proteins in the Ten‐4 ‐/‐ tissue. 14‐3‐3s act as regulatory factors in diverse intracellular signaling pathways, including the suppression of apoptosis by regulating mitochondrial permeability (Stevers et al. 2018). Therefore, we hereafter focused on the expression and function of 14‐3‐3s.

**TABLE 1:** Downregulated proteins expressed in the Ten‐4 ‐/‐ dorsal column.

No	Score	Gene	Protein	Accession
1	4349.39	Mbp	Myelin basic protein	P04370‐4
2	4073.98	Mbp	Myelin basic protein	Q542T4
3	837.54	Tubb4a	Tubulin beta‐4A chain	Q9D6F9
4	456.07	Cnp	2′,3′‐cyclic‐nucleotide 3′‐phosphodiesterase	Q3TYL9
5	280.44	Plp1	Myelin proteolipid protein	Q3UYM8
6	157.78	Sirt2	NAD‐dependent protein deacetylase	Q3UJK6

*Note:* Proteins, whose scores are more than 100 and abundance ratios of Ten‐4 ‐/‐ to WT are less than 0.8, are listed by the label‐free quantification of mass‐spectrometry analysis results. The score and abundance ratio were calculated by the search engine SequestHT.

**TABLE 2:** Upregulated proteins expressed in the Ten‐4 ‐/‐ dorsal column.

No	Score	Gene	Protein	Accession
1	1308.87	Nefm	Neurofilament, medium polypeptide	A0A0R4J036
2	1297.88	Nefl	Neurofilament, light polypeptide	P08551
3	743.66	Nefh	Neurofilament, heavy polypeptide	P19246
4	552.72	Actb	ACTB protein	Q3UAF6
5	501.44	Atp1a2	Sodium/potassium‐transporting ATPase subunit alpha (Fragment)	Q6ZQ49
6	466.42	Atp5f1b	ATP synthase subunit beta, mitochondrial	P56480
7	465.50	Gm10358	Glyceraldehyde‐3‐phosphate dehydrogenase	A0A1D5RLD8
8	426.28	Ina	Alpha‐internexin	P46660
9	386.81	Slc25a4	ADP/ATP translocase 1	P48962
10	342.42	Atp5f1a	ATP synthase subunit alpha	Q03265
11	265.36	Eno2	Phosphopyruvate hydratase	Q545V3
12	222.49	Aldoa	Fructose‐bisphosphate aldolase	Q5FWB7
13	192.59	Hbbt1	Beta‐globin	A8DUK7
14	190.87	Ywhaq	14‐3‐3 protein gamma subtype	A8IP69
15	181.74	Sptan1	Spna2 protein	B9EKJ1
16	181.41	Uchl1	Ubiquitin carboxyl‐terminal hydrolase isozyme L1	Q9R0P9
17	178.74	Ywhaz	14‐3‐3 protein zeta/delta	P63101
18	173.51	Vim	IF rod domain‐containing protein	Q3U6S1
19	165.22	Aldoc	Fructose‐bisphosphate aldolase C	P05063
20	159.82	Mdh2	Malate dehydrogenase, mitochondrial	P08249
21	156.98	Calm2	Calmoduin‐2	P0DP27
22	153.59	Hba‐a1	Alpha globin 1	Q91VB8
23	152.67	Glud1	Glutamate dehydrogenase 1, mitochondrial	P26443
24	151.16	Prph	Peripherin	P15331
25	144.14	Stxbp1	Syntaxin‐binding protein 1	O08599
26	133.11	Ywhae	14‐3‐3 protein epsilon	P62259
27	128.13	Hspd1	60 kDa heat shock protein, mitochondrial	P63038
28	122.79	Ldhb	l‐lactate dehydrogenase B chain	P16125
29	121.06	Aco2	Aconitate hydratase, mitochondrial	Q99KI0
30	121.00	Slc25a5	ADP/ATP translocase 2	P51881
31	119.06	Ywhab	14‐3‐3 protein beta/alpha	Q9CQV8

*Note:* Proteins, whose scores are more than 100 and abundance ratios of Ten‐4 ‐/‐ to WT are more than 1.2, are listed by the label‐free quantification of mass‐spectrometry analysis results. The score and abundance ratio were calculated by the search engine SequestHT.

### 3.4 Elevated Expression of 14‐3‐3s in Ten‐4 ‐/‐ CST Axons

**FIGURE 6:**
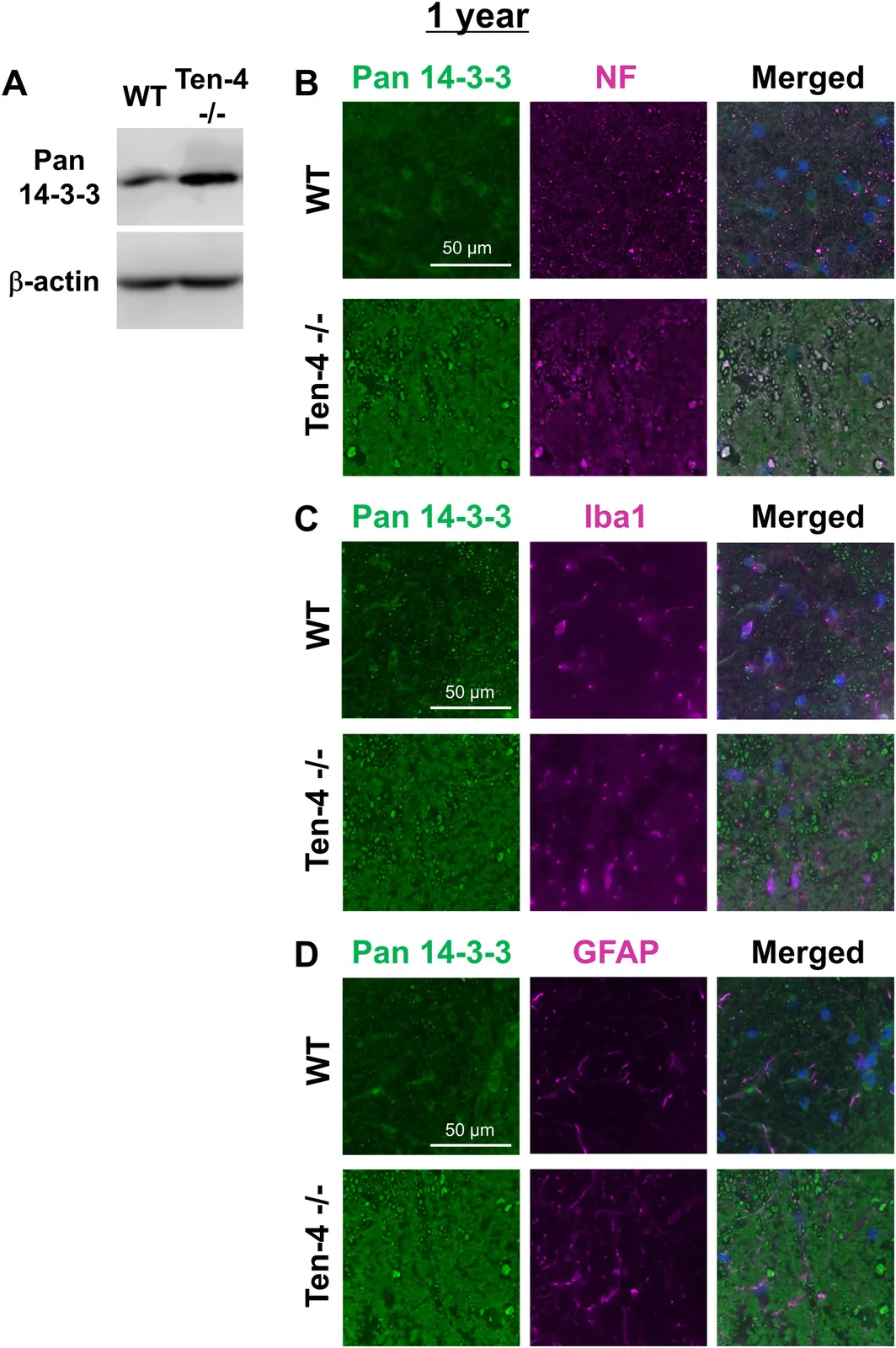
Expression of 14‐3‐3s in the Ten‐4 ‐/‐ mouse DC tissue. (A) Western blotting of 14‐3‐3 and β‐actin from 1‐year‐old WT and Ten‐4 ‐/‐ DCs. Immunostaining of pan 14‐3‐3 and markers: NF (B), Iba1 (C), and GFAP (D) in CSTs of 1‐year‐old WT and Ten‐4 ‐/‐ mice. DAPI was used to stain nuclei. Scale bar: 50 μm.

Western blotting using samples of 1‐year‐old DC tissues and anti‐pan 14‐3‐3 antibody showed that 14‐3‐3s expression was upregulated in the Ten‐4 ‐/‐ DC (Figure 6A). Next, to examine the localization of 14‐3‐3s, we performed immunohistochemical staining in the CST of 1‐year‐old mice. The expression of 14‐3‐3s was elevated in the CST of Ten‐4 ‐/‐ mice, compared with WT (Figure 6B–D). The elevated expression of 14‐3‐3s and NFs, which were dotted or diffusely stained, was obviously overlapped in the Ten‐4 ‐/‐ CST, indicating that 14‐3‐3 is upregulated in small‐diameter axons in the CST of Ten‐4 ‐/‐ mice (Figure 6B). In contrast, little signal of 14‐3‐3s upregulated in Ten‐4 ‐/‐ CSTs was merged with Iba1 and GFAP, while the weaker expression of 14‐3‐3s was detected in these glial cells in the WT CST (Figure 6C,D). It is noted that the number of cells labeled by DAPI was lower in the Ten‐4 ‐/‐ CST, compared with WT, as the number of OLs is substantially reduced in the Ten‐4 ‐/‐ CST (Hayashi, Suzuki, Takahashi, et al. 2020).

### 3.5 Progression of the Axonal Damage by Inhibition of 14‐3‐3s

**FIGURE 7:**
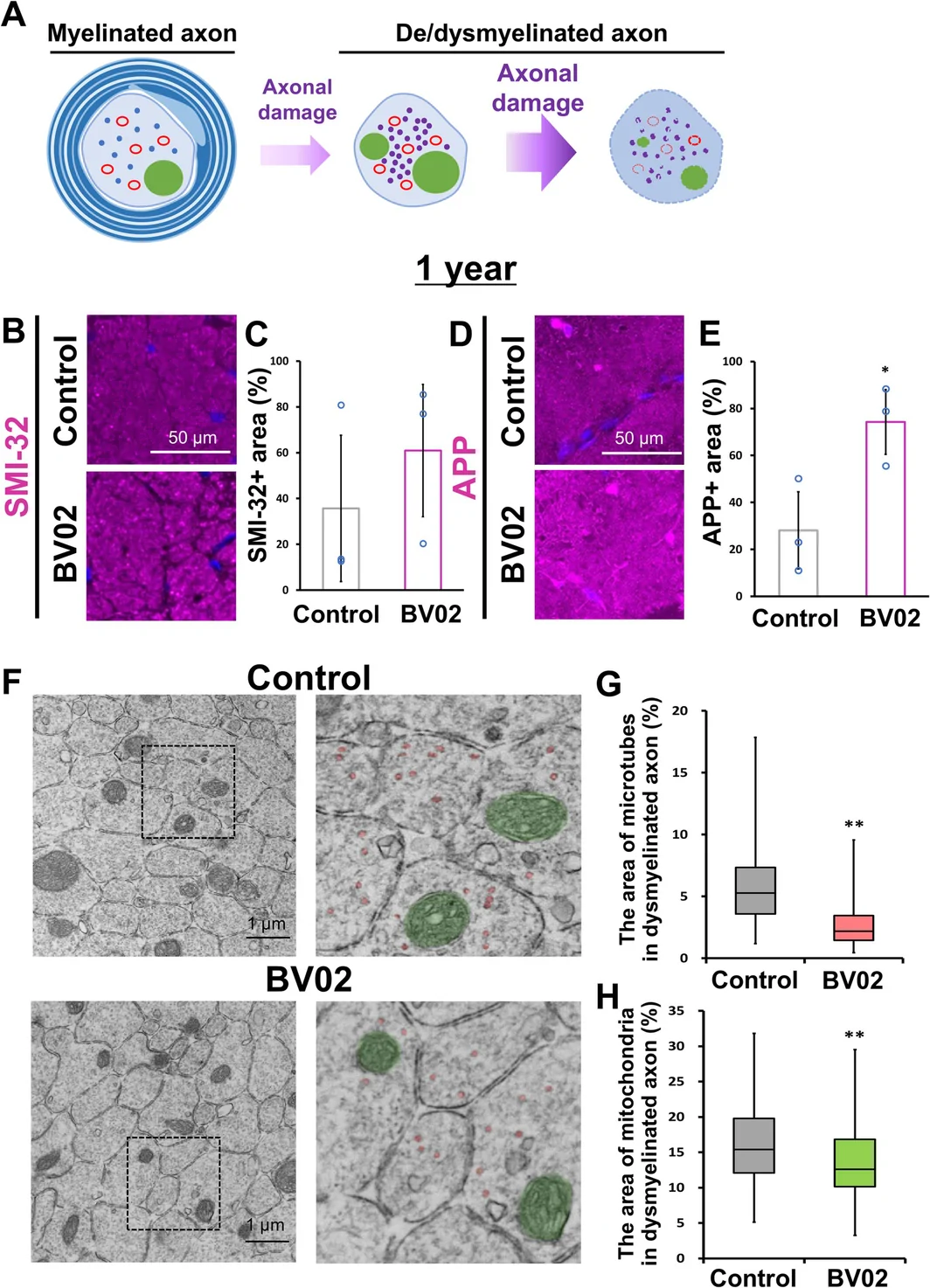
Progression of the damage in dysmyelinated axons by inhibition of 14‐3‐3s with BV02. (A) Schematic representation of axon structure. Axonal cytoskeletons, such as MTs (red circles) and NFs (phosphorylated NFs: blue dots; dephosphorylated NFs: purple dots), and mitochondria (green) are organized in a myelinated axon. Loss of myelin by de‐ or dysmyelination leads to a decrease in NF spacing at the beginning of axonal damage. At the late stage of the damage, the number and sizes of cytoskeletons, including MTs, and mitochondria are decreased. Immunohistochemical images of non‐phosphorylated NF (SMI‐32) (B) and APP (D) in BV02‐treated or only solvent (DMSO)‐treated CST tissues of Ten‐4 ‐/‐ mice. DAPI was used to stain nuclei. Scale bar: 50 μm. Quantifications of SMI‐32‐positive (C) and APP‐positive (E) areas in the CST tissues. Three individual mouse tissues were analyzed (*p < 0.05, Student's *t*‐test, error bars represent mean ± SD). (F) TEM images and quantification of the area of (G) MTs and (H) mitochondria in dysmyelinated axons in the CST of Ten‐4 ‐/‐ mice. Green: Mitochondria, Red: MT. One hundred independent axons from three individual mouse CSTs were analyzed (**p < 0.01, Student's *t*‐test, Box plots represent the median, interquartile ranges (box) and minimum/maximum values (whiskers)). Scale bar: 1 μm.

To investigate the role of 14‐3‐3s as a pro‐survival factor of dysmyelinated axons, we first performed an inhibition experiment using BV02, a chemical inhibitor of 14‐3‐3s. Ten‐4 ‐/‐ mice were intraperitoneally injected with BV02 or only its solvent as a control every day for 7 days. Spinal cords of these mice were dissected out for immunochemical and ultrastructural analyses. As a result of immunohistochemistry of SMI‐32 and APP, we found that APP signal was significantly increased in the CST tissue treated with BV02, compared to its control, while in SMI‐32 staining, a statistical difference was not observed (Figure 7B–E). Since a decrease in the number and sizes of MTs and mitochondria is observed at the late stage of damage/degeneration (Figure 7A) (Trapp and Stys 2009; Dutta and Trapp 2011), we additionally measured and quantified the area of MTs and mitochondria per axon (Figure 7F; in red and green, respectively). The quantitative analysis showed that the area of MTs and mitochondria was significantly reduced in BV02‐treated axons (Figure 7G,H). These results indicated that BV02 promoted the progression of the damage in dysmyelinated small‐diameter axons.

**FIGURE 8:**
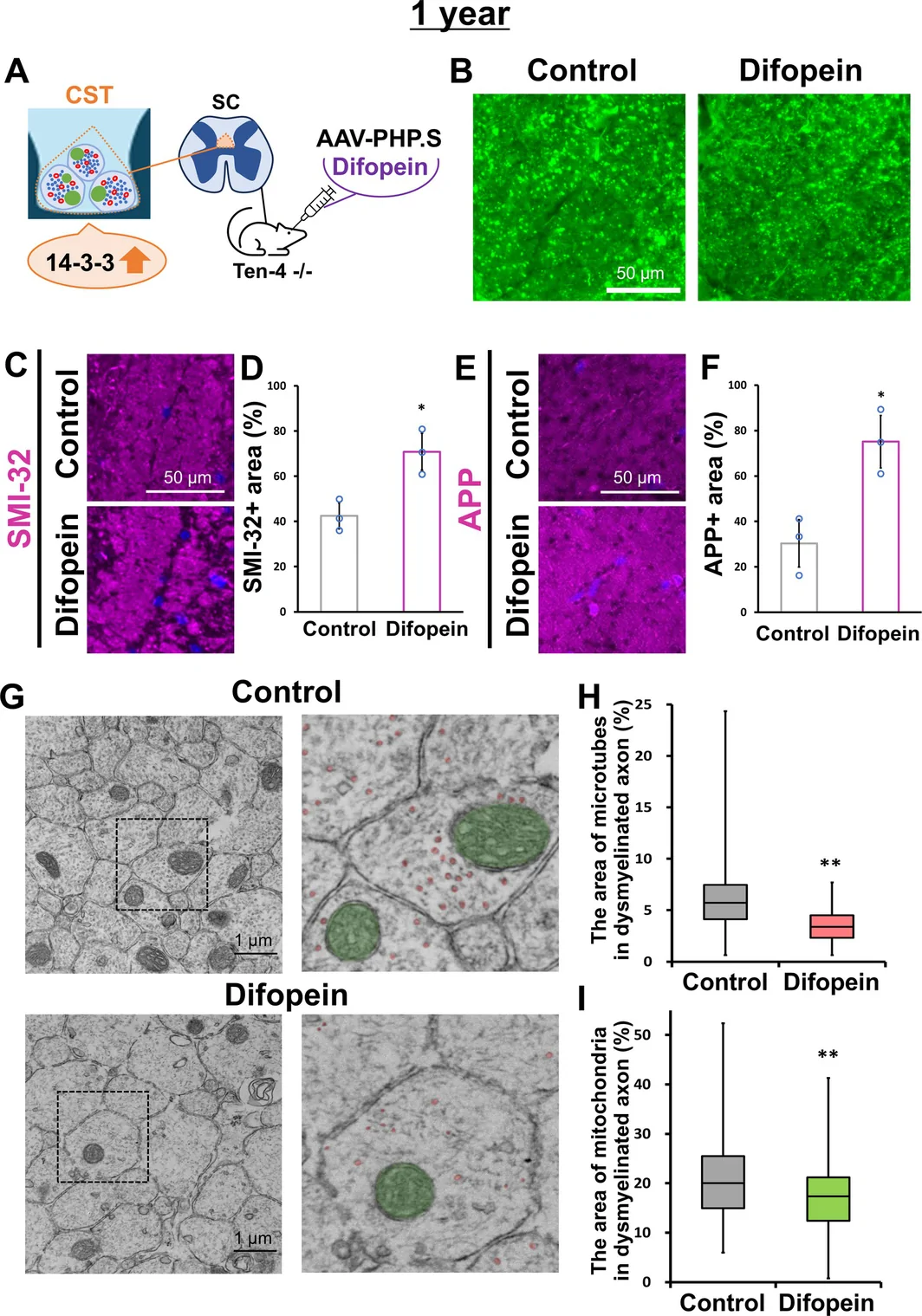
Progression of the damage of dysmyelinated axons by inhibition of 14‐3‐3s with difopein. (A) Experimental scheme. (B) Immunohistochemical images of EGFP in the CSTs of Ten‐4 ‐/‐ mice that were injected AAV‐PHP.S‐difopein or its control. Most of small‐diameter axons in the CSTs were positive for EGFP. Scale bar: 50 μm. Immunohistochemical images of nonphosphorylated NF (SMI‐32) (C) and APP (E) in difopein‐expressed or its control CST tissues of Ten‐4 ‐/‐ mice. DAPI was used to stain nuclei. Scale bar: 50 μm. Quantifications of SMI‐32‐positive (D) and APP‐positive (F) areas in the CST tissues. Three individual mouse tissues were analyzed (*p < 0.05, Student's *t*‐test, error bars represent mean ± SD). (G) TEM images and quantification of the area of (H) MTs and (I) mitochondria in dysmyelinated axon in the CST of Ten‐4 ‐/‐ mice. Green: mitochondria, Red: MT. One hundred independent axons from three individual mouse CSTs were analyzed (**p < 0.01, Student's *t*‐test. Box plots represent the median, interquartile ranges (box) and minimum/maximum values (whiskers)). Scale bar: 1 μm.

We further analyzed 14‐3‐3s function by the more specific method using the other 14‐3‐3 inhibitor difopein expressed under the synapsin‐1 promoter, which is specifically driven in neurons. Also, we used AAV‐PHP.S, which is blood–brain barrier‐permeable and highly infects into the dorsal root ganglia and the spinal cord tissue, as a carrier for the expression of difopein (Figure 8A). Ten‐4 ‐/‐ mice were intravenously administered AAV‐PHP.S carrying the difopein gene or without the gene of difopein as a control. After 3 weeks, spinal cords of these mice were dissected out and immunochemical and ultrastructural analyses were performed. The infection of the AAVs in the spinal cord CST was confirmed by the expression of EGFP, which was encoded in both the difopein‐ and control‐plasmids (Figure 8B). Immunohistochemical analysis of SMI‐32 and APP revealed that both staining signals were significantly higher in the difopein‐expressed CST than the control (Figure 8C–F). We then analyzed the ultrastructure of dysmyelinated axons in the CST by EM (Figure 8G). As a result, a significant decrease was observed in the percentage of the axoplasm area occupied by either MTs or mitochondria (Figure 8H,I). In summary, the inhibitions of 14‐3‐3s function promoted the axonal damage, indicating that 14‐3‐3s are among the pro‐survival factors of dysmyelinated small‐diameter axons.

## 4 Discussion

Axon degeneration due to myelin defects has been studied in several mutant mice. Knockout mice of PLP, CNP, and monocarboxylate transporter (MCT) 1 develop axonal degeneration at the age of 8–14 months, while myelin is normally formed in these mice (Griffiths et al. 1998; Lappe‐Siefke et al. 2003; Lee et al. 2012). The deficiencies of these proteins interfere with the transportation systems of nutrients from OL myelin to axons, as the formation of the cytoplasmic channels and the supply of monocarboxylates (Lee et al. 2012; Snaidero et al. 2014). In contrast, severe dysmyelination in *shiverer* mice and myelin regulatory factor (Myrf) knockout mice leads to short life spans and does not allow analysis of dysmyelinated axons in the long term (Chernoff 1981; Emery et al. 2009). In this study, we used Ten‐4 ‐/‐ mice, whose OL myelination is extensively inhibited, but survive for longer than a year (Suzuki et al. 2012). Moreover, the dysmyelination in Ten‐4 ‐/‐ mice is observed specifically around small‐diameter axons, which are dominantly damaged in MS (DeLuca et al. 2004; Suzuki et al. 2012). Therefore, this mouse line is unique and our findings in this study are useful for the characterization of the maintenance of dysmyelinated small‐diameter axons in the long term.

**FIGURE 9:**
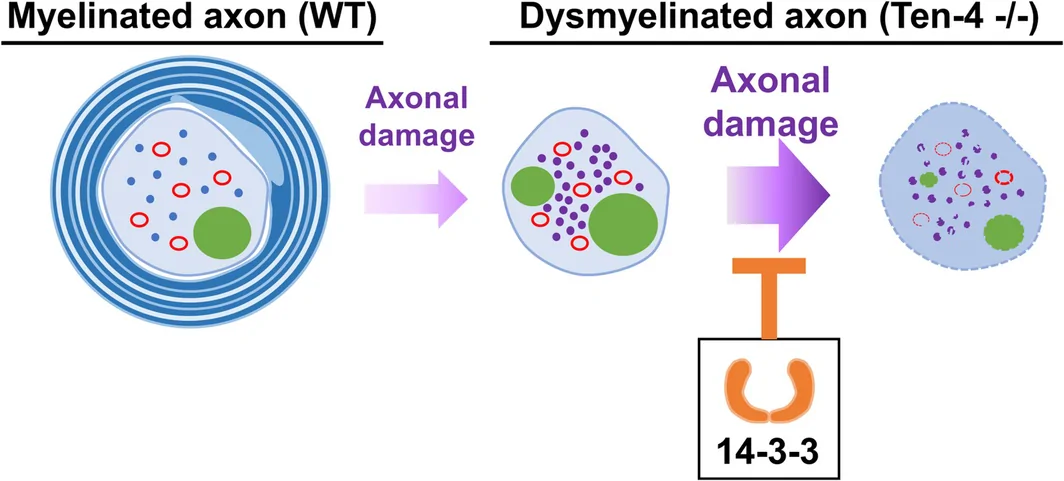
Schematic diagram of the maintenance of dysmyelinated axons in Ten‐4 ‐/‐ mice. The early stage of the damage is observed in dysmyelinated small‐diameter axons in Ten‐4 ‐/‐ mouse tissues. The high expression of 14‐3‐3s arrests the entrance of the late stage of the damage, which results in the survival of the dysmyelinated small‐diameter axons.

In normal myelinated axons, NFs are phosphorylated and distributed at a certain distance from each other. At the onset of damage/degeneration, dephosphorylation of NFs occurs, followed by spacing of closely between dephosphorylated NFs. The NFs are more or less fragmented at this stage (Dutta and Trapp 2011). These fragmented NFs were more detectable in Ten‐4 ‐/‐ tissues, compared with WT, by the immunohistochemistry and mass spectrometry in the present study. Also, microglia and astroglia likely responded to the damage as a secondary reaction in Ten‐4 ‐/‐ mice. However, we observed the maintenance of the aged Ten‐4 ‐/‐ axons with increases in the number of mitochondria and the expression of the proteins for ATP production pathways, indicating that these axons survived by arresting the entrance into the late stage of the damage, which leads to degeneration (Figure 9) (Mahad et al. 2009). As a result of the inhibition of 14‐3‐3s, the immunohistochemical signal of APP and the dephosphorylation of NFs were increased. In addition, the reduction of MTs and mitochondria was observed, which is typically detected at the late stage of axonal damage/degeneration (Figure 9) (Dutta and Trapp 2011). From these aspects, 14‐3‐3s play a role in the survival of dysmyelinated axons. Furthermore, the increase of 14‐3‐3 proteins is detected in the cerebrospinal fluid of MS patients, which implies feedback of the damage in demyelinated axons (Colucci et al. 2004). Interestingly, a recent report indicates that demyelinated axons in MS and experimental autoimmune encephalomyelitis (EAE) mice are more tolerant to degeneration than myelinated axons (Schäffner et al. 2023). In these cases, dysfunctional myelin acts as a physical barrier that does not allow axons to take up metabolites from the extracellular space. In addition to the removal of dysfunctional myelin, the activity of the pro‐survival factors, such as 14‐3‐3s, may be required for the axons to be maintained.

Due to 14‐3‐3s binding to a variety of signaling proteins, they regulate various cellular processes. One of the important pathways is apoptotic signaling. The interaction of 14‐3‐3s with phosphorylated client proteins, such as Bcl2‐associated agonist of cell death (Bad), Bcl2‐associated X protein (Bax), and Bcl2‐interacting mediator of cell death (Bim), positively regulates anti‐apoptotic signals (Stevers et al. 2018). When these proteins are dephosphorylated, they penetrate into mitochondria, and cytochrome *c* in the mitochondrial inner membrane is then released by them, which results in the inhibition of the electron transfer system for ATP production (Garrido et al. 2006). This triggers cell apoptosis. Therefore, the anti‐apoptotic signaling mediated by high expression of 14‐3‐3s may be activated to maintain dysmyelinated axons. Fusicoccin‐A (FC‐A) is a stabilizer of the 14‐3‐3 binding to client proteins and is positively effective on axon regeneration (Kaplan et al. 2017). It is possible that FC‐A enhances axon survival in demyelination.

Another question is why these are/were observed specifically in small‐diameter axons. In MS and Pelizaeus‐Merzbacher disease, small‐diameter axons in the CST and FG are more vulnerable (Garbern et al. 2002; DeLuca et al. 2004). Here, Ten‐4 ‐/‐ mice also showed damage in the small‐diameter axon regions, especially the CST and FG. Edgar et al. demonstrated that the distal ends of long axons in the FG are most susceptible to degeneration, because of myelin dysfunction in PLP mutant mice (Edgar et al. 2004). In the Ten‐4 ‐/‐ spinal cord, Type I/II OLs that preferentially myelinate small‐diameter axons are not differentiated (Hayashi, Suzuki, Takahashi, and Akazawa 2020). From these observations, the vulnerability of small‐diameter axons is presumably due to the long axonal distance from their soma and the distinct functions of OL subpopulations. Further analyses are required to elucidate the mechanisms.

Taken together, in Ten‐4 ‐/‐ mice, small‐diameter axons are damaged after dysmyelination, but are maintained. The survival of dysmyelinated small‐diameter axons is positively regulated by 14‐3‐3s. Our findings will be useful for better understanding of the normal and pathological mechanisms in axon maintenance, and for developing a therapeutic reagent to inhibit the progression or improve the symptoms in the myelin‐related diseases as MS.

## Author Contributions

M.S. and N.S. designed the experiments. N.Y., H.S., R.T., M.F., Y.H., B.S., M.Y., C.H., T.J.M., S.B., and N.S. performed experiments. N.Y., H.S., R.T., A.Y., and R.O. analyzed data. H.S., T.J.M., F.M.B., and N.S. prepared resources. N.Y., B.S., S.A., and N.S. wrote the manuscript. N.S. supervised this work. All the authors read and approved the final manuscript.

## Funding

This work was supported by the Ministry of Education, Culture, Sports, Science, and Technology (20K07756, 24K10486, and 20KK0188) and Sumitomo Foundation (200490).

## Conflicts of Interest

The authors declare no conflicts of interest.

## Supporting information


**Figure S1:** Three different regions in the white matter dorsal column.

**Figure S2:** Representative SEM images for the quantification analyses of EM data.

**Figure S3:** EM analysis of myelin and axon structures at 7 weeks.

**Data S1:** Supporting Information figure captions.

## Data Availability

The data that supports the findings of this study are available in the Supporting Information of this article.
