# Supplementary material for: The Maintenance of Dysmyelinated Small‐Diameter Axons by 14‐3‐3s in the Central Nervous System
Source: Glia. 2026 Sep 9;74(11):e70221. doi: 10.1002/glia.70221 (PMC13556523; doi:10.1002/glia.70221)
Supplement: Supplementary file 1 [file GLIA-74-0-s004.zip › GLIA-70221-Sup-0004-Supinfo1.docx]

Supplementary Information

The maintenance of dysmyelinated small-diameter axons by 14-3-3s in the central nervous system

Nanako Yamada^1#^, Hinako Saito^1,2,3#^, Riko Takahashi^2^, Mio Fujisato^1^, Yukina Hosoda^2^, Binri Sasaki^1^, Momona Yamada^2^, Sakurako Abe^1^, Chikako Hayashi^2^, Akira Yoshimoto^1^, Ryunosuke Ohkawa^1^, Tomal Julian Matt^4,5,6^, Florence Bareyre^4,5,6^, Stefan Berghoff^7,8^, Mikael Simons^7,8^, and Nobuharu Suzuki^1,2*^

^1^Department of Clinical Bioanalysis and Molecular Biology, Graduate School of Medical and Dental Sciences, Institute of Science Tokyo/Tokyo Medical and Dental University (TMDU), Tokyo, Japan

^2^Department of Molecular and Cellular Biology, Graduate School of Medical and Dental Sciences, TMDU, Tokyo, Japan

^3^Division of Surgical Pathology, Institute of Science Tokyo Hospital, Tokyo, Japan

^4^Institute of Clinical Neuroimmunology, University Hospital, LMU Munich, Munich, Germany.

^5^Biomedical Center Munich, Medical Faculty, LMU Munich, Planegg-Martinsried, Munich, Germany.

^6^Munich Cluster of Systems Neurology, Munich, Germany.

^7^Institute of Neuronal Cell Biology, Technical University Munich, Munich, Germany.

^8^German Center for Neurodegenerative Diseases, Munich, Germany.

^*^To whom correspondence should be addressed: Nobuharu Suzuki, Ph.D., Department of Clinical Bioanalysis and Molecular Biology, Graduate School of Medical and Dental Sciences, Institute of Science Tokyo/TMDU, 1-5-45 Yushima, Bldg 3, Bunkyo-ku, Tokyo, Japan, 113-8510, E-mail: [nsuzbb@tmd.ac.jp](mailto:nsuzbb@tmd.ac.jp), Tel: +81-3-5803-5364; Fax: +81-3-5803-5364.

^#^These authors equally contributed to this work.

**Supplementary Fig. S1:** Three different regions in the white matter dorsal column.

**Supplementary Fig. S2:** Representative SEM images for the quantification analyses of EM data.

**Supplementary Fig. S3:** EM analysis of myelin and axon structures at 7 weeks.
